# Prediction of Adverse Events in Stable Non-Variceal Gastrointestinal Bleeding Using Machine Learning

**DOI:** 10.3390/jcm9082603

**Published:** 2020-08-11

**Authors:** Dong-Woo Seo, Hahn Yi, Beomhee Park, Youn-Jung Kim, Dae Ho Jung, Ilsang Woo, Chang Hwan Sohn, Byuk Sung Ko, Namkug Kim, Won Young Kim

**Affiliations:** 1 Department of Emergency Medicine, College of Medicine, University of Ulsan, Asan Medical Center, Seoul 05505, Korea; leiseo@gmail.com (D.-W.S.); dbswjdsla@gmail.com (Y.-J.K.); faradays520@gmail.com (D.H.J.); schwan97@gmail.com (C.H.S.); 2 Department of Information Medicine, College of Medicine, University of Ulsan, Asan Medical Center, Seoul 05505, Korea; 3 Asan Institute for Life Sciences, Asan Medical Center, Seoul 05505, Korea; hahn.yi@gmail.com; 4 Department of Convergence Medicine, University of Ulsan College of Medicine, Asan Medical Center, Seoul 05505, Korea; qkr18x@gmail.com (B.P.); bidfore@gmail.com (I.W.); 5 Department of Emergency Medicine, College of Medicine, Hanyang University, Seoul 04763, Korea; postwinston@gmail.com

**Keywords:** emergency departments, machine learning, upper gastrointestinal bleeding, mortality, hypotension, endoscopy

## Abstract

Clinical risk-scoring systems are important for identifying patients with upper gastrointestinal bleeding (UGIB) who are at a high risk of hemodynamic instability. We developed an algorithm that predicts adverse events in patients with initially stable non-variceal UGIB using machine learning (ML). Using prospective observational registry, 1439 out of 3363 consecutive patients were enrolled. Primary outcomes included adverse events such as mortality, hypotension, and rebleeding within 7 days. Four machine learning algorithms, namely, logistic regression with regularization (LR), random forest classifier (RF), gradient boosting classifier (GB), and voting classifier (VC), were compared with the Glasgow–Blatchford score (GBS) and Rockall scores. The RF model showed the highest accuracies and significant improvement over conventional methods for predicting mortality (area under the curve: RF 0.917 vs. GBS 0.710), but the performance of the VC model was best in hypotension (VC 0.757 vs. GBS 0.668) and rebleeding within 7 days (VC 0.733 vs. GBS 0.694). Clinically significant variables including blood urea nitrogen, albumin, hemoglobin, platelet, prothrombin time, age, and lactate were identified by the global feature importance analysis. These results suggest that ML models will be useful early predictive tools for identifying high-risk patients with initially stable non-variceal UGIB admitted at an emergency department.

## 1. Introduction

Although the morbidity and mortality rates of upper gastrointestinal bleeding (UGIB) have decreased recently, this condition remains a burden to public health, with a mortality rate of 6–12% and hospital cost of more than $2.5 billion yearly in the United States [1,2]. Therefore, current guidelines recommend early risk stratification of patients with non-variceal UGIB to identify high- or low-risk patients in order to help in decision-making, including timing of endoscopy, disposition (admission vs. outpatient), and level of care (general ward vs. intensive care unit) [3]. 

Several scoring systems such as the Glasgow–Blatchford score (GBS) and the Rockall score [4,5] have been developed for assessing patients with UGIB; however, they have limitations in detecting high-risk patients with UGIB who require endoscopy, embolization, or surgical treatment and who have higher risk of developing hemodynamic instability [6,7,8,9]. Moreover, the subjectivity of the definitions of hepatic disease and cardiac disease included in the GBS, given its complexity of calculation and requirement for laboratory results or endoscopic assessment, makes its application in clinical practice difficult [1,9,10]. 

Machine learning (ML) is a discipline that uses computational modeling to learn from data, showing that executing a specific task improves with experience [11,12,13,14,15]. Thus, ML models may improve upon the risk stratification provided by existing clinical risk assessment tools and help guide clinical decision-making. However, studies of ML models in UGIB are limited by relatively small sample sizes (all but two studies had <1000 patients) or identification of low-risk patients; therefore, further evidence is needed [16,17]. 

Early identification of high-risk UGIB is an integral component in patient admission at emergency rooms. ML models, which can use more variables than are possible with conventional clinical scores, can improve decision-making regarding the timing of intervention or treatment option. This study aimed to develop and validate an algorithm that predicts adverse events such as mortality, hypotension, and rebleeding in patients with initially stable suspected non-variceal UGIB using several ML algorithms.

## 2. Experimental Section

### 2.1. Data

The UGIB registry was collected in the Emergency Department (ED) of the Asan Medical Center, which has a census record of 110,000 visits per year and serves as a tertiary referral center in Seoul, Korea. The study period was from January 1, 2012 to April 30, 2017. All consecutive patients older than 18 years visiting the ED with suspected non-variceal UGIB during the study period were included in the registry. 

UGIB was defined by the chief complaints of hematemesis, coffee-ground color vomiting, or melena [1,9,18]. Patients who had liver cirrhosis with variceal bleeding or advanced neoplasm were excluded. Patients who were already in a hypotensive state, defined by systolic blood pressure (SBP) of <90 mmHg at admission to the ED, were also excluded; we focused on the development of a model for initially normotensive patients with non-variceal UGIB. Our institutional review board approved the study and waived the requirement for informed consent.

The variables used in this study included demographics, initial vital signs, comorbidities, mental change, syncope, fresh blood in the nasogastric tube, melena on rectal examination, specific medications that could cause gastrointestinal bleeding (non-steroidal anti-inflammatory drugs, antiplatelet agents, anticoagulants), hemoglobin level, platelet count, prothrombin time, international normalized ratio, blood urea nitrogen level, creatinine level, albumin level, base deficit, lactate level, GBS, pre-endoscopy Rockall score, lactate, and hypotension development [1].

Nasogastric tube and rectal examinations were performed in all patients with UGIB except those who refused. Patients with shock, clinical deterioration, or hemoglobin less than 7 g/dL were considered for the blood transfusion. Transfusion decisions for coagulopathy or thrombocytopenia were determined by the treating physician considering comorbidities. Pre-endoscopic intravenous proton pump inhibitors were used in all patients with UGIB until there was no evidence of peptic ulcer disease. Except in cases of refusal, we conducted endoscopy within 24 h in all patients with UGIB. The mental status of patients was classified by the AVPU (alert, voice, pain, unresponsive) scale. If fresh blood was found in a nasogastric tube after 500 mL of manual irrigation, the case was defined as positive. Chronic liver diseases included chronic hepatitis B, hepatitis C, alcoholism, autoimmune disease, or others. An advanced neoplasm was defined as a neoplasm by distant metastasis. After the first endoscopy, rebleeding was suspected when the patient showed unstable vital signs, melena, hematochezia, hematemesis, or a decrease in hemoglobin of 2 g/dL or more. Rebleeding was confirmed when active bleeding or a fresh blood clot was found in the endoscopy of suspected rebleeding patients. 

The primary outcome was adverse events, including in-hospital mortality, the development of hypotension within 24 h of ED admission (which was characterized by SBP < 90 mmHg without other causes of hypotension except for UGIB), and rebleeding within 7 days [1,9].

### 2.2. Methods (Machine Learning Algorithms)

Four ML models were developed by 5-fold cross-validation: logistic regression with regularization (LR), random forest classifier (RF), gradient boosting classifier (GB), and voting classifier (VC). A 5-fold cross-validation might prevent the generation of overfitted models by avoiding cases that biased the test-sets due to imbalanced single-hospital data. The models predicted whether a patient would experience mortality, hypotension, or rebleeding within 7 days by learning 38 variables, which are categorized by demographics, comorbidities, associated symptom signs, initial vital signs, and laboratory findings. A grid-search with cross-validation was used to tune the hyperparameters of the ML models. The grid-search method determined the optimal hyperparameters of the models from user-defined grids of initial parameters. The grid-search method also helped the ML models to determine the global optimal hyperparameters without falling into local maximums. We set the grid-search method to optimize the area under the receiver operating characteristic curve of cross-validation sets. 

Logistic regression is one of statistical models to classify categorical outcomes [19]. Logistic regression can estimate the probability of outcomes as a function of many input variables. In particular, LR has shown better performance on unseen data than logistic regression without regularization. Because of its easy interpretability and familiarity, it was used as a baseline model to evaluate the performance of other ML methods. RF is an algorithm that predicts the outcome by voting of trained decision tree models for numerous randomly sampled data [20]. RF is suitable for our analysis because it predicts outcomes robustly even if we input a large number of non-scaled features that are not relevant to predicting the outcome. GB starts with a simple model and continually adds more enhanced models described by the residuals of a previous model using gradient information [21]. RF is a bagging machine that is made up of independent decision trees, while GB is a boosting learner that combines decision trees constructed sequentially. Therefore, GB is considered a more accurate predictor than RF, but it tends to overfit a training dataset. Lastly, VC is a machine that classifies outcomes by selecting the majority of results from several ML models [22]. In our case, VC was composed of LR, RF, and GB at a ratio of 1:1:1. 

### 2.3. Analysis

Continuous variables were expressed as the median with interquartile range (IQR). Categorical variables were reported as numbers and percentages. The Mann-Whitney U test or Fisher’s exact test was used to compare the values of continuous variables. The Chi-square test was applied for categorical variables. For all the reports, a two-sided *p*-value of <0.05 was considered statistically significant. The area under the curve (AUC) was used to evaluate the performance of the models; the Brier score and the logarithmic loss were also calculated to provide a nuanced view of the accuracy of the models. The mean values and 95% confidence intervals of all measurements were calculated using 7 iterations with 7 different random seeds which were fixed for reproducibility. The global feature importance was calculated by the degree of score drop in the model by randomly shuffling the one single variable sequentially. Local interpretable model-agnostic explanation (LIME) was introduced to interpret the outcome predictions of individual patients [23]. LIME is a way to approximate a complex non-linear model to a linear model near variables of interest for improving human understanding, instead of trying to explain the global working principle of the models. LIME works by sampling new input variables, similar to variables of our subjects, and investigating which variable causes the predictive probability to change significantly. ML analysis and plotting were performed using an open-source program language (Python 3.7.1) and its packages (numpy-1.16.1, scikit-learn 0.20.3, imblearn 0.4.3, lime 0.1.1.33, matplotlib 3.0.2).code. 

## 3. Results

### 3.1. Baseline Statistics

To develop models predicting mortality, hypotension, and rebleeding within 7 days for initially normotensive patients with non-variceal UGIB, data were collected from 1439 patients who met the appropriate screening criteria. In this study, we excluded 1038 patients with known liver cirrhosis with variceal bleeding, 519 patients who were already in a hypotensive state, 313 patients with advanced neoplasm, and 54 patients with missing values. The baseline statistics of the variables for the class groups of mortality, hypotension, and rebleeding within 7 days are summarized in Table 1. The baseline statistics of the variables for the included patients are presented in Table S1.

### 3.2. Model Performances

**Figure 1:**
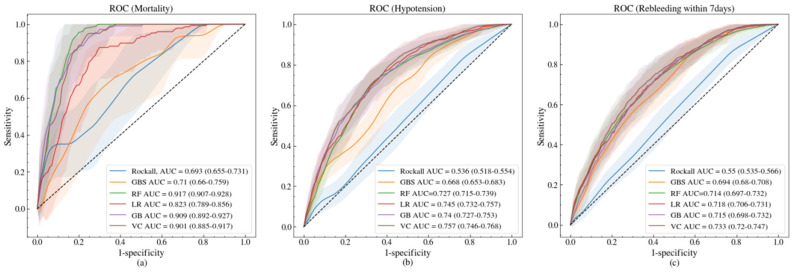
Receiver operating characteristic (ROC) and AUC of Rockall (blue), GBS (Glasgow–Blatchford score, orange), LR (logistic regression with regularization, red), RF (random forest classifier, green), GB (gradient boosting classifier, light purple), and VC (voting classifier, light brown) for mortality (**a**), hypotension (**b**), and rebleeding within 7 days (**c**), from left to right. The shaded regions stand for 1 standard deviation from the mean of the ROC curves. Mean AUC and its 95% confidence interval of the models are shown in the legends of the subplots.

The AUC for the Rockall score and GBS was 0.536 and 0.668 for hypotension, 0.693 and 0.710 for mortality, and 0.550 and 0.694 for rebleeding within 7 days, respectively. The Rockall score and GBS in the ML models for predicting these three outcomes are shown in Figure 1. All ML models for the three outcomes outperformed the Rockall score and GBS in terms of the AUC. For mortality, the AUC of the machine learning models was significantly different from that of Rockall score and GBS by 0.113–0.224, but the improvement of ML for both hypotension and rebleeding within 7 days was relatively smaller: 0.059–0.221 and 0.020–0.183, respectively. 

The performance of the six models for three outcomes was compared by predicting numbers of patients, the AUC, Brier score, and log loss, as shown in Table 2. In all four scorings, the performance was largely improved in the order of Rockall score, GBS, LR, RF, VC, and GB. The predicted ratio of positive patients was higher than the observed ratio of those even in the GB model because many normal patients had false-positives, which is very severe when the outcome is mortality. The log loss of mortality, an outcome with a severe data imbalance, was greater than 0.1; thus, the log loss was a non-informative metric. Only log loss of the GB models for hypotension and rebleeding within 7 days were less than 0.33, better than that of a random classifier, considering the data imbalance with the ratio of the two classes being 1:10. For imbalanced data like our study data, the Brier loss, which focuses on the minority class, is more adequate for scoring the predicted probabilities. When comparing the Brier skill score, which is computed from the Brier score, and the Brier reference score, all models predicting mortality failed to perform better than a random classifier. The RF, GB, and VC models predicting hypotension and rebleeding within 7 days, however, were effective in prognosticating patients. The prediction result of a test-set by the VC is shown in the confusion matrix (Table S2). In addition, the predicted number of outcomes, sensitivity, specificity, positive predictive value, negative predictive value, AUC, and their 95% confidence intervals were computed to measure the performance of the model dependence on the threshold values (Table S3).

### 3.3. Model Validation

**Figure 2:**
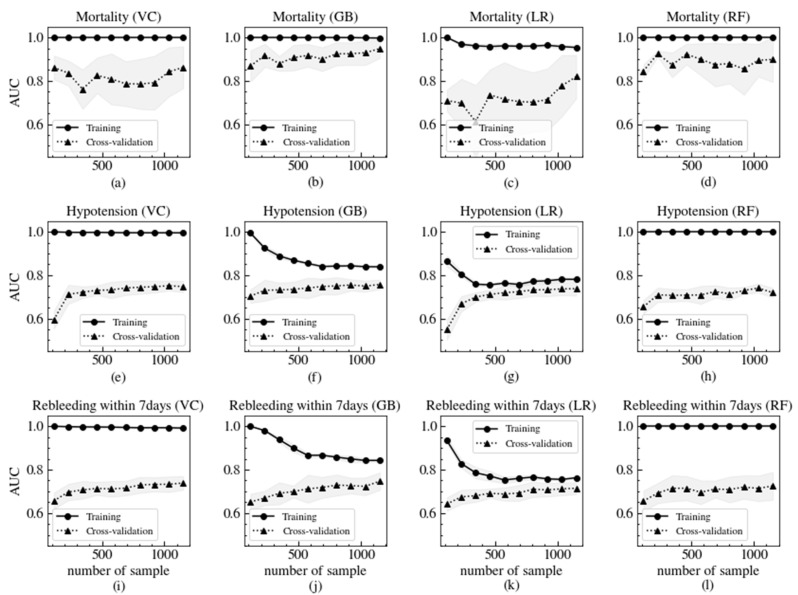
According to the number of sample, the AUC learning curves of VC (**a**), GB (**b**), LR (**c**) and RF (**d**) for mortality. When the outcome is hypotension, (**e**), (**f**), (**g**) and (**h**) are the AUC learning curves as a function of the number of samples of VC, GB, LR and RF respectively. The AUC learning curves of VC, GB, LR and RF for rebleeding within 7 days are shown in (**i**), (**j**), (**k**) and (**l**) respectively. Circles and solid lines symbolize the learning curve of the mean AUC for the training data-set. Triangles and dotted lines represent the learning curve of the mean AUC for the test data-set. The 1 standard deviation ranges are shown in the shaded region of the markers and line types.

In Figure 2, the AUCs according to the number of samples are plotted to determine whether the models were overfitting and we had enough data to train the ML models. The models predicting both hypotension and rebleeding within 7 days seemed to be fully trained, but more data made it possible to improve the models’ prediction of mortality, especially in the GB model. The RF models seemed to have overfit the training dataset because the AUC of the training dataset was equal to 1 regardless of the number of samples and the test dataset showed slight improvement. In the learning curve studies with all predicting variables, RF classifiers were readily overfitting with a small number of samples. Therefore, more samples are needed for training classifiers with the RF algorithm.

**Figure 3:**
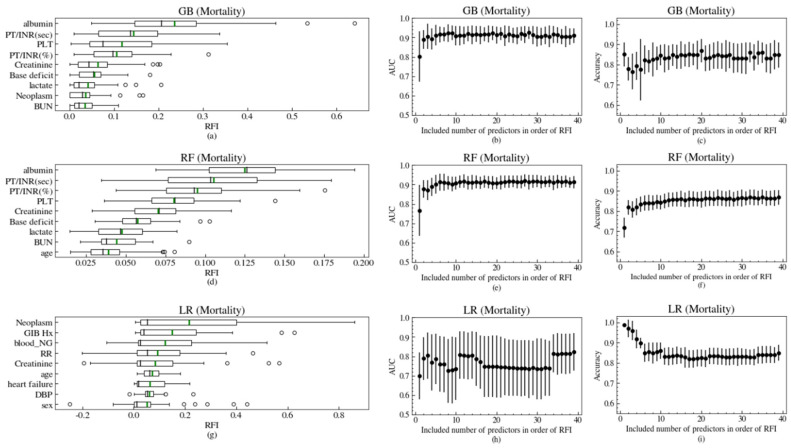
(**a**), (**d**), and (**g**) are the relative feature importance (RFI) of the top nine features predicting mortality by the three machine learning algorithms: LR, RF, and GB respectively. The black lines in boxes show the median of RFI and green lines show the mean of RFI. The AUC of the three models, with the included number of predictors in the order of RFI to predict mortality, are plotted in (**b**), (**e**), and (**h**). The accuracies of the three models, according to the included number of predictors in the order of RFI to predict mortality, are also plotted in (**c**), (**f**), and (**i**). The means of the AUC and accuracy are shown as black dots and the 1 standard deviation ranges from them are shown as black error-bars. See Table A1 for variable names.

The feature selection process was conducted to select useful variables to predict the outcomes and ensure that the variables matched the intuition from the clinical experiences. We only examined the relative features importance (RFI) of three classifiers except the VC in mortality (Figure 3), hypotension (Figure S1), and rebleeding within 7 days (Figure S2). For computing the AUC and the accuracy of models for mortality, the variables were added one by one from the largest RFI to the smallest one in Figure 3. The RFI of the LR model had a large standard deviation due to the fact that some folds of the training dataset did not converge properly. Because of some commonalities in the algorithm, there were common important variables for the GB and LF models. Although the order of RFI in the LR models was different from that in the GB and LF models, many significant variables were found in the top nine of the models’ RFI for all three of the ML algorithm models. It was confirmed that the larger the computed AUC, the more predictive variables the model contained (Figure 3b,e,h). However, the accuracy of LR was shown as an L-shaped curve as the number of independent variables of the model increased. This is because the LR model was inclined to predict actual positives (poor outcomes) as false-negatives (good outcomes), which has been a common overfitting problem in training models from imbalance data. The accuracy of a GB algorithm model with very few features might have an overfitting problem, but it would not be as severe as that of the LR models. The accuracy of the RF models, which is good at avoiding the problem of predicting almost-positive outcomes as negative ones, is enhanced by an increasing number of included variables in the model.

**Figure 4:**
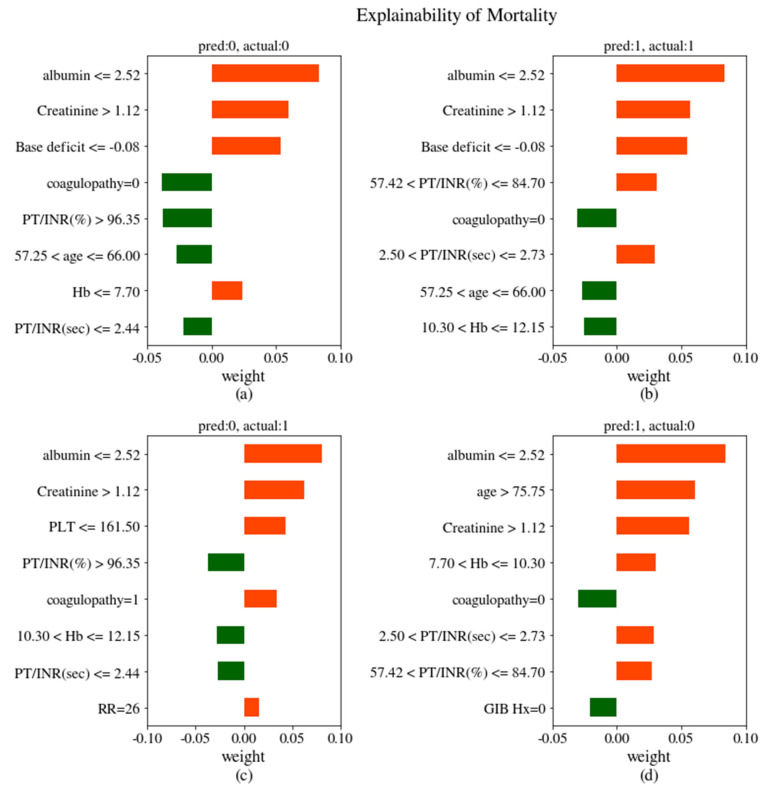
LIME (local interpretable model-agnostic explanation) explanation of true-negative (**a**), true-positive (**b**), false-negative (**c**), and false-positive (**d**) cases for the RF when the outcome is mortality. The orange bars are the variables that support the positive and the green bars are the variables supporting the negative. The larger the absolute value of weight, the greater the effect of the predicting outcome, mortality. See Table A1 for variable names.

The cases of true-negatives, true-positives, false-positives, and false-negatives by the RF were used to explain the prediction of mortality with LIME interpretation in Figure 4. The four cases were selected from the test-set randomly. Only the eight most important features in predicting mortality for an individual patient were shown for simplicity. In cases of true-negatives (Figure 4a), the values of coagulopathy, prothrombin ratio/international normalized ratio (PT/INR) (sec), age, and PT/INR (%) supported a low probability of mortality in the patients, but the values of albumin, creatinine, base deficit, and hemoglobin supported the positive prediction. In the case of true-positives, the values of albumin, creatinine, base deficit, PT/INR (%), and PT/INR (sec) in Figure 4b support a high chance of mortality of the patients. In cases of false-negatives (Figure 4c), it looked vague for predicting the possibility of mortality with a distribution of eight feature values. The values of hemoglobin, PT/INR (sec), and PT/INR (%) contradict the risk of mortality of the patients, but the values of albumin, creatinine, platelet count, coagulopathy, and respiratory rate denied the decision. The last case (Figure 4d) showed that the values of all features except coagulopathy and previous gastrointestinal history explained the high risk of mortality, but it actually did not as it was a false-positive. Despite this false-positive case, the RF seemed to have made the right decision for mortality of patients based on the predicted variable distribution. Almost statistically significant variables for the mortality of patients appeared again in the LIME explanation diagram. The four cases of LIME explainability of hypotension and rebleeding within 7 days are shown in Figures S3 and S4.

## 4. Discussion

In this study, we demonstrated that ML approaches using prospectively collected high-quality GIB datasets (*n* = 1439) provide better diagnostic capacity for the detection of high-risk patients associated with hypotension, rebleeding, and mortality than conventional clinical risk scoring systems. 

Among patients with UGIB, early identification of high-risk patients may help provide appropriate intervention, thus reducing mortality and morbidity. However, the primary use of risk scores in clinical practice that are recommended by guidelines is the identification of very low-risk patients for outpatient management [3,8,24]. The optimal score for the detection of low-risk patients who can be safely discharged from the ED without early endoscopy was shown in published studies as a GBS of 0 or 1 [3,24]. Although a recent large prospective study by Stanley et al. demonstrated that a GBS score of more than 7 could be an indicator of a need for endoscopic intervention, the sensitivity and specificity were not enough (80.4% and 57.4%, respectively) [18]. This is consistent with our results of a relatively low AUC for GBS and Rockall score. 

Our study demonstrated the superior performance of risk stratification for stable patients with suspected UGIB using an ML approach, which has several applications and advantages. First, an ML approach can be applicable using electronic health record derived data. Given the vast amount of data currently being accumulated in electric health records (EHR), the importance of ML is expected to increase. In other clinical areas, EHR is already used to deploy an ML approach for decision support [25,26]. Second, an ML approach can use more variables than is possible with conventional clinical scores. Third, it can be applied quickly in the clinical field by immediately learning from data collected in real time. Learning through real-time data can show good results over time. Fourth, the patients’ emergency room data used in our study were not extracted under well-designed research like a randomized controlled trial. An ML approach is good for generalized pattern recognition, classification, and prediction, even using data with complex features [27]. 

Previous studies using an ML approach for UGIB had limitations such as a small sample size or a risk of bias from a heterogeneous dataset. In this field, the use of ML approaches is still in its infancy. Rotondano et al. demonstrated a superior performance over the Rockall score for 1-month mortality using a neural network model [28]. However, that study did not compare other well-known risk scores like GBS. Moreover, several studies using predictive models had different subjects [29,30]. 

Recently, Shung et al. developed a ML model that compared clinical risk scoring systems [16]. That study, like our study, attempted ML using data from a large sample. It had the advantage of external validation including multi-racial patients. However, an exact comparison is difficult because our study targeted only patients who came to the emergency room. The data collection period of our study, 5 years, is relatively long, so the bias for time in our data is less than in that study. We presented global feature importance results so that clinicians can compare them with their integrated clinical experience of UGIB. A way to explain how this model predicted the adverse events of individual patients with UGIB was also introduced. More emphasis will be placed on the predictive abilities of models as the use of ML applications expands in medicine. To the best of our knowledge, our study is the first to use ML to classify the risk of UGIB in patients admitted to an emergency room.

There are several limitations to our study. Although our study has the advantage of having a large sample size, the data collected from a single hospital seemed to be subject to overfitting and limitations of generalization. Other excellent scoring systems, such as AIMS65, PNED (progetto nazionale emorragia digestive), and HARBINGER (Horibe gAstRointestinal BleedING scoRe), were not compared in this study [18,31]. To be able to compare, we considered whether there were many citations and excellent performance when selecting the scoring system [18]. We thought that comparing GBS and Rockall score with our ML model could concisely show the purpose of the paper. In future studies, it is necessary to build a machine learning model that can be compared to other scoring systems. Table-type data from emergency rooms are prone to having insufficient information for predicting a patient’s adverse events. The variables of tabular data can provide the averaged status or snapshot information of a patient. The power of predicting patients’ adverse events in our model was also affected by this limitation. Because of the small number of deaths, there is little confidence in the construction of the model by artificial intelligence. The data used in our study are considered imbalanced data, which is common in the field of medical research. Imbalanced data occur when there is a skewed distribution of class representations: generally, several negative samples and a few positive samples. The problem was especially severe when the outcome was mortality in this study. To overcome the problems caused by the imbalanced data, various techniques such as a data-level method, algorithm-level method, and their combination were suggested [32]. In our studies, data resampling methods, combining random under-sampling of a majority group [33] and Tomek’s link [34], were only used to predict mortality. Class weight arguments, inverse proportion to positive and negative class frequencies, and adaptation of classifier’s thresholds were commonly applied to estimate all three outcomes.

## 5. Conclusions

A new approach using ML algorithms showed a higher detection ability of adverse events, namely mortality, hypotension, and rebleeding, in patients with high-risk UGIB. These results suggest that ML models can be a predictive tool for early identification of high-risk patients with initially stable non-variceal UGIB admitted at an ED.

## Figures and Tables

**Table 1:** Statistical characteristics of the two groups divided by the mortality, hypotension, and rebleeding within 7 days classes in the study population.

Characteristic	Mortality			Hypotension			Rebleeding within 7 days		
	No (*n* = 1422)	Yes (*n* = 17)	*p*-Value	No (*n* = 1297)	Yes (*n* = 142)	*p*-Value	No (*n* = 1305)	Yes (*n* = 134)	*p*-Value
Demographics									
Age	63 (52–74)	75 (59–80)	0.011	64 (52–74)	63 (54–73)	0.900	64 (52–74)	64.5 (56–76)	0.062
Male	426 (30.0)	7 (41.2)	0.461	405 (31.2)	28 (19.7)	0.006	401 (30.7)	32 (23.9)	0.122
Comorbidities									
Diabetes mellitus	304 (21.4)	5 (29.4)	0.614	269 (20.7)	40 (28.2)	0.052	276 (21.1)	33 (24.6)	0.410
Hypertension	538 (37.8)	4 (23.5)	0.338	490 (37.8)	52 (36.6)	0.857	488 (37.4)	54 (40.3)	0.571
Cardiac disease	313 (22.0)	5 (29.4)	0.662	286 (22.1)	32 (22.5)	0.980	296 (22.7)	22 (16.4)	0.120
Liver disease	64 (4.5)	1 (5.9)	0.753	60 (4.6)	5 (3.5)	0.697	55 (4.2)	10 (7.5)	0.132
Coagulopathy	267(18.8)	9 (52.9)	0.001	239 (18.4)	37 (26.1)	0.038	235 (18.0)	41 (30.6)	<0.001
Ischemic heart disease	188 (13.2)	3 (17.6)	0.861	174 (13.4)	17 (13.2)	0.725	179 (13.7)	10 (9.0)	0.158
Heart failure	52 (3.7)	3 (17.6)	0.019	46 (3.5)	9 (6.3)	0.157	50 (3.8)	5 (3.7)	0.858
Neoplasm	187 (13.2)	9 (52.9)	<0.001	168 (13.0)	28 (19.7)	0.036	165 (12.6)	31 (23.1)	0.001
Chronic kidney disease	123 (8.6)	4 (23.5)	0.086	118 (9.0)	9 (6.3)	0.345	114 (8.7)	13 (9.7)	0.829
Previous GIB history	229 (16.1)	8 (47.1)	0.002	209 (16.1)	28 (19.7)	0.327	210 (16.1)	27 (20.1)	0.279
COPD	29 (2.0)	1 (5.9)	0.804	27 (2.1)	3 (2.1)	0.776	27 (2.1)	3 (2.2)	0.852
Stroke	126 (8.9)	1 (5.9)	0.999	117 (9.0)	10 (7.0)	0.527	118 (9.0)	9 (6.7)	0.457
Associated symptom and signs									
Syncope	52 (3.7)	1 (5.9)	0.870	43 (3.3)	10 (7.0)	0.045	43 (3.3)	10 (7.5)	0.028
Hematemesis	500 (35.2)	8 (47.1)	0.444	449 (34.6)	59 (41.5)	0.122	449 (34.4)	59 (44.0)	0.034
Melena, chief complaint	922 (64.8)	9 (52.9)	0.444	848 (65.4)	83 (58.5)	0.122	856 (63.6)	75 (56.0)	0.034
Melena on rectal examination	650 (45.7)	8 (47.1)	0.893	571 (44.0)	87 (61.3)	<0.001	576 (44.1)	82 (61.2)	<0.001
Fresh blood on nasogastric tube	155 (10.9)	5 (29.4)	0.043	125 (9.6)	35 (24.6)	<0.001	127 (9.8)	33 (24.6)	<0.001
Mental change	29 (2.0)	4 (23.5)	<0.001	28 (2.2)	5 (3.5)	0.463	29 (3.2)	4 (3.0)	0.796
Drug history Antiplatelet agent	275 (19.3)	0 (0.0)	0.088	255 (19.7)	20 (14.1)	0.136	259 (19.8)	16 (11.9)	0.036
NSAIDs	47 (3.3)	0 (0.0)	0.940	44 (3.3)	3 (2.1)	0.571	45 (3.4)	2 (1.5)	0.338
Anticoagulation	103 (7.2)	2 (11.8)	0.808	91 (7.0)	14 (9.9)	0.286	92 (7.0)	13 (9.7)	0.342
Vital signs									
SBP (mmHg)	123 (111–141)	118 (110–130)	0.534	125 (112–142)	109 (101–118)	<0.001	124 (111–141)	116 (107–132)	<0.001
DBP (mmHg)	75 (67–86)	72 (62–86)	0.703	76 (68–86)	68 (62–77)	<0.001	76 (68–86)	73 (65–81)	0.009
Heart rate (/min)	89 (76–103)	100 (86–118)	0.048	89 (76–103)	90 (77–107)	0.229	89 (76–103)	92 (79–105)	0.149
Respiratory rate (/min)	20 (18–20)	20 (20–20)	0.045	20 (18–20)	20 (18.5–20)	0.229	20 (18–20)	20 (20–20)	0.331
Body temperature (°C)	36.5 (36.2–36.8)	36.5 (36–36.9)	0.835	36.5 (36.2–36.9)	36.4 (36–36.6)	<0.001	36.5 (36.2–36.9)	36.5 (36–36.8)	0.187
Saturation of peripheral oxygen, room air (%)	99 (97–100)	99 (96–99)	0.325	99 (97–100)	99 (97–100)	0.880	99 (97–100)	99 (98–100)	0.851
Laboratory findings									
Hemoglobin (g/dL)	10.3 (8.3–12.5)	8.5 (7.2–10.3)	0.018	10.4 (8.4–12.6)	9.4 (7.4–11.5)	<0.001	10.5 (8.4–12.6)	8.7 (7.2–10.6)	<0.001
Platelet count (×10^3^/mm^3^)	223 (169–276)	104 (69–239)	0.011	223 (169–277)	211 (160–260)	0.209	225 (170–277)	191 (153–260)	0.002
PT/INR (%)	90.9 (79.2–103)	62.9 (51.6–78.6)	<0.001	91.0 (79.3–103.2)	88.1 (70–97.4)	0.003	91.2 (79.5–103)	85.8 (70.3–97.4)	<0.001
PT/INR (s)	2.47 (2.42–2.54)	2.7 (2.6–2.8)	<0.001	2.47 (2.42–2.54)	2.5 (2.4–2.6)	0.004	2.47 (2.42–2.54)	2.5 (2.4–2.6)	0.001
BUN (mg/dL)	24.0 (15.0–38.0)	35.0 (24.0–62.0)	0.006	23.0 (15.0–37.0)	31.0 (22.0–43.0)	<0.001	23.0 (15.0–37.0)	31.0 (22.0–45.8)	<0.001
Creatinine (mg/dL)	0.8 (0.7–1.1)	1.2 (1.0–2.9)	0.015	0.8 (0.7–1.1)	0.9 (0.7–1.2)	0.156	0.0 (0.0–0.8 (0.7–1.1)1.0)	0.9 (0.7–1.2)	0.134
Albumin (g/dL)	3.3 (2.8–3.7)	2.3 (1.9–2.5)	<0.001	3.3 (2.9–3.8)	3.0 (2.5–3.4)	<0.001	3.4 (2.9–3.8)	2.8 (2.3–3.3)	<0.001
Lactate (mmol/L)	1.3 (0.9–2.1)	2.2 (1.1–5.7)	0.051	1.3 (0.9–2.0)	1.6 (1.2–2.6)	<0.001	1.3 (0.9–2.0)	1.6 (1.1–2.4)	0.013
Base deficit (mmol/L)	1.5 (−0.5–3.5)	−0.9 (−6.8–6.0)	0.433	1.7 (−0.5–3.7)	0.8 (−1.7–2.8)	<0.001	1.7 (−0.5–3.6)	0.5 (−0.8–2.8)	0.002
Risk scores									
Glasgow-Blatchford Bleeding Score	10 (6–12)	13 (11–14)	0.003	10 (6–12)	12 (9–14)	<0.001	10 (6–12)	12 (10–14)	<0.001
Pre-endoscopy Rockall	1 (1–3)	2 (1–6)	0.005	1 (1–3)	1 (1–3)	0.147	1 (1–3)	2 (1–3)	0.049

Data are presented as median (interquartile range) and number (percentage), GIB (gastrointestinal bleeding), COPD (chronic obstructive pulmonary disease), NSAIDs (non-steroidal anti-inflammatory drugs), SBP (systolic blood pressure), DBP (diastolic blood pressure), PT (prothrombin time), INR (international normalized ratio), BUN (blood urea nitrogen).

**Table 2:** The performance of models for predicting mortality, hypotension, and rebleeding within 7 days evaluated by the predicted positive ratio, AUC, brier score, and log loss.

Outcomes	Models Scorings	Rockall Score	GBS	Logistic Regression Classifier	Random Forest Classifier	Gradient Boosting Classifier	Voting Classifier	Actual Positive Ratio (%)
Mortality	Predicted Positive ratio (%, 95% CI)	29.3 (28.4–30.2)	25.2 (24.4–26.0)	14.7 (13.4–15.9)	14.6 (13.5–15.6)	15.4 (13.8–17.0)	16.5 (15.8–17.1)	1.18
	AUC (95% CI)	0.694 (0.649–0.738)	0.715 (0.676–0.754)	0.826 (0.792–0.861)	0.909 (0.896–0.921)	0.911 (0.893–0.930)	0.908 (0.893–0.922)	
	Brier score (95% CI)	0.294 (0.285–0.303)	0.250 (0.242–0.258)	0.129 (0.120–0.139)	0.034 (0.032–0.036)	0.035 (0.032–0.039)	0.050 (0.047–0.054)	
	Log loss (95% CI)	27.0 (26.7–27.3)	34.1 (34.0–34.1)	0.510 (0.435–0.563)	0.142 (0.137–0.147)	0.126 (0.116–0.137)	0.180 (0.171–0.189)	
Hypotension	Predicted Positive ratio (%, 95% CI)	45.0 (44.1–45.9)	35.0 (34.1–35.8)	26.1 (25.1–27.2)	22.56 (21.52–23.59)	16.8 (15.4–18.2)	19.7 (18.7–20.8)	9.87
	AUC (95% CI)	0.536 (0.516–0.555)	0.668 (0.656–0.680)	0.747 (0.737–0.758)	0.739 (0.727–0.751)	0.756 (0.743–0.768)	0.766 (0.755–0.777)	
	Brier score (95% CI)	0.233 (0.226 0.240)	0.273 (0.267–0.279)	0.194 (0.191–0.198)	0.0834 (0.0826–0.08416)	0.082 (0.081–0.084)	0.092 (0.091–0.093)	
	Log loss (95% CI)	25.0 (24.8–25.3)	31.1 (31.0–31.1)	0.569 (0.559–0.579)	0.329 (0.310–0.348)	0.289 (0.283–0.295)	0.319 (0.316–0.322)	
Rebleeding within 7 days	Predicted Positive ratio (%, 95% CI)	45.0 (44.3 45.8)	35.0 (33.8 36.1)	31.6 (30.6 32.7)	27.4 (26.2 28.5)	20.7 (19.0 22.3)	21.3 (20.2 22.4)	9.31
	AUC (95% CI)	0.550 (0.534–0.566)	0.694 (0.680–0.708)	0.712 (0.698–0.726)	0.707 (0.689–0.725)	0.717 (0.699–0.734)	0.729 (0.714–0.745)	
	Brier score (95% CI)	0.223 (0.215–0.231)	0.258 (0.249–0.266)	0.201 (0.197–0.206)	0.081 (0.080–0.082)	0.081 (0.080–0.082)	0.092 (0.090–0.093)	
	Log loss (95% CI)	25.0 (24.8–25.2)	31.3 (31.2–31.3)	0.589 (0.578–0.599)	0.319 (0.295–0.344)	0.287 (0.281–0.293)	0.323 (0.319–0.327)	

## Supplementary Materials

The following are available online at https://www.mdpi.com/2077-0383/9/8/2603/s1, **Table S1**: The baseline statistics in all variables for patients included in the study, **Table S2**. Confusion matrices for a test-set data (n = 287) by the voting classifier with the threshold for mortality, hypotension, and rebleeding within 7 days, **Table S3**. The number of outcomes, sensitivity, specificity, PPV, NPV, and AUC for a test-set data by the 6 models with various thresholds in three outcomes, mortality, hypotension, and rebleeding within 7 days, **Figure S1**: The relative feature importance (RFI) of the top 9 features predicting hypotension, and both AUC and accuracy as functions of included number of predictors in descending order of RFI, **Figure S2**: The relative feature importance (RFI) of the top 9 features predicting rebleeding within 7 days, and both AUC and accuracy as functions of included number of predictors in descending order of RFI, **Figure S3**: LIME of four cases for VC when outcome is hypotension, **Figure S4**: LIME of four cases for VC when outcome is rebleeding within 7 days.

## Appendix A

**Table A1:** (left) and those in model training and figures (right).

Official Variable Name	Convenient Variable Name
Demographics	
Age	age
Male:1	sex
Comorbidities	
Diabetes Mellitus	DM
Hypertension	HTN
Cardiac disease	cardiac disease
Liver disease	liver disease
Coagulopathy	coagulopathy
Ischemic heart disease	IHD
Heart failure	heart failure
Neoplasm	Neoplasm
Chronic kidney disease	CKD
Previous GIB history	GIB Hx
COPD	COPD
Stroke	stroke
Associated symptom and signs	
Syncope	syncope
Hematemesis	Hematemesis
Melena, chief complaint	Melena
Melena on rectal examination	melena
Fresh blood on nasogastric tube	blood NG
Mental change	mental binary
Drug history Antiplatelet agent	offend antiplatelet
NSAIDs	offend NSAID
Anticoagulation	offend anticoagulation
Vital signs	
SBP (mmHg)	SBP
DBP (mmHg)	DBP
Heart rate (/min)	HR
Respiratory rate (/min)	RR
Body temperature (°C)	BT
Saturation of peripheral oxygen (%)	SpO2
Laboratory findings	
Hemoglobin (g/dL)	Hb
Platelet count (×10^3^/mm^3^)	PLT
PT/INR (%)	PT/INR (%)
PT/INR (s)	PT/INR (sec)
BUN (mg/dL)	BUN
Creatinine (mg/dL)	creatinine
Albumin (g/dL)	albumin
Lactate (mmol/L)	lactate
Base deficit (mmol/L)	Base deficit
