# Supplementary material for: Prediction of Adverse Events in Stable Non-Variceal Gastrointestinal Bleeding Using Machine Learning
Source: J Clin Med. 2020 Aug 11;9(8):2603. doi: 10.3390/jcm9082603 (PMC7464777; doi:10.3390/jcm9082603)
Supplement: Supplementary file 1 [file jcm-09-02603-s001.pdf]

Table S1. The baseline statistics in all variables for patients included in the study.

**Demographic**	**Total (N=1,439)**
Age, median (IQR)	64 (52–74)
Sex: male, n (%)	1,006 (69.9)
Comorbidities	
Diabetes Mellitus: 1, n (%)	309 (21.5)
Hypertension: 1, n (%)	542 (37.7)
Cardiac disease: 1, n (%)	318 (22.1)
Liver disease: 1, n (%)	65 (4.52)
Coagulopathy: 1, n (%)	276 (19.2)
Ischemic heart disease: 1, n (%)	191 (13.3)
Heart failure: 1, n (%)	55 (3.8)
Neoplasm: 1, n (%)	196 (13.6)
Chronic kidney disease: 1, n (%)	127 (8.8)
Previous GIB history: 1, n (%)	237 (16.5)
COPD: 1, n (%)	30 (2.1)
Stroke: 1, n (%)	127 (8.8)
Associated symptom and signs	
Syncope: 1, n (%)	53 (3.7)
Hematemesis: 1, n (%)	508 (35.3)
Melena, chief complaint: 1, n (%)	931 (64.7)
Melena on rectal examination: 1, n (%)	658 (45.7)
Fresh blood on nasogastric tube: 1, n (%)	160 (11.1)
Mental change: 1, n (%)	33 (2.3)
Drug history Antiplatelet agent: 1, n (%)	275 (19.1)
NSAIDs: 1, n (%)	47 (3.3)
Anticoagulation: 1, n (%)	105 (7.3)
Vital signs	
SBP (mmHg), median (IQR)	123 (111–141)
DBP (mmHg), median (IQR)	75 (67–86)
Heart rate (/min), median (IQR)	89 (76–103)
Respiratory rate (/min), median (IQR)	20 (18–20)
Body temperature ([illegible]), median (IQR)	36.5 (36.2–36.8)
Saturation of peripheral oxygen (%), median (IQR)	99.0 (97.0–100)
Laboratory findings	
Hemoglobin (g/dL), median (IQR)	10.3 (8.2–12.4)
Platelet count (×10^3 /mm^3), median (IQR)	222 (168–276)
PT/INR (%), median (IQR)	90 (78–102)
PT/INR (s), median (IQR)	2.47 (2.42–2.54)
BUN (mg/dL), median (IQR)	24 (15–38)

Creatinine (mg/dL), median (IQR)	0.85 (0.69–1.08)
Albumin (g/dL), median (IQR)	3.3 (2.8–3.7)
Lactate (mmol/L), median (IQR)	1.3 (0.9–2.1)
Base deficit (mmol/L), mean ± SD	1.5 (-0.6–3.6)
Risk scores	
Glasgow-Blatchford Bleeding Score, median (IQR)	10 (6–3)
Pre-endoscopy Rockall, median (IQR)	1 (1–3)
Outcomes	
Mortality, n (%)	17 (1.2)
Hypotension, n (%)	142 (9.9)
Rebleeding within 7 days, n (%)	134 (9.3)

Data are presented as median (interquartile range) and number (percentage).

Table S2. Confusion matrices for a test-set data (n=287) by the voting classifier with the threshold for mortality, hypotension, and rebleeding within 7 days.

**Mortality (threshold = 0.34)**			
	Prediction -	Prediction +	Total
Actual -	250	34	284
Actual +	0	3	3
Total	250	37	287
**Hypotension (threshold = 0.31)**			
	Prediction -	Prediction +	Total
Actual -	223	36	259
Actual +	13	15	28
Total	236	51	287
**Rebleeding within 7 days (0.29)**			
	Prediction -	Prediction +	Total
Actual -	197	64	261
Actual +	9	17	26
Total	206	81	287

Table S3. The number of outcomes, sensitivity, specificity, PPV, NPV, and AUC for a test-set data by the 6 models with various thresholds in three outcomes, mortality, hypotension, and rebleeding within 7 days.

Classifiers	Mortality (N = 287)						
	Cut-off threshold	Number of outcomes	Sensitivity	Specificity	PPV	NPV	AUC
Pre–endoscopy Rockall score	1	228 (226–231)	100 (100–100)	1.49 (1.42–1.56)	20.9 (20.1–21.7)	100 (100–100)	0.621 (0.607–0.635)
	2	130 (127–132)	99.4 (99.2–99.5)	1.86 (1.64–2.08)	55.3 (54.3–56.2)	70.5 (63.1–77.9)	0.622 (0.609–0.635)
	3	84.4 (81.8–87.0)	99.1 (99.0–99.3)	1.91 (1.53–2.28)	70.9 (70.0–71.8)	47.4 (38.7–56.0)	0.619 (0.606–0.631)
	4	48.2 (46.1–50.3)	99.1 (98.9–99.2)	2.48 (1.80–3.15)	83.5 (82.7–84.2)	35.2 (25.7–44.7)	0.616 (0.604–0.628)
Glasgow–Blatchford Bleeding Score	10	158 (156–161)	99.6 (99.4–99.7)	1.76 (1.58–1.95)	45.3 (44.3–46.3)	82.1 (74.5–89.8)	0.573 (0.549–0.597)
	11	129 (126–134)	99.5 (99.4–99.6)	2.01 (1.82–2.21)	55.5 (54.5–56.4)	76.7 (69.6–83.7)	0.602 (0.581–0.623)
	12	100 (98.3–103)	99.5 (99.3–99.6)	2.39 (2.12–2.66)	65.5 (64.7–66.3)	71.0 (63.1–78.8)	0.622 (0.603–0.641)
	13	72.6 (70.4–74.8)	99.4 (99.2–99.5)	2.78 (2.38–3.19)	75.2 (74.4–76.0)	59.3 (50.8–67.7)	0.632 (0.615–0.650)
	14	40.2 (38.2–42.2)	99.0 (98.9–99.1)	2.55 (1.95–3.16)	86.2 (85.5–86.9)	30.0 (23.2–36.8)	0.624 (0.608–0.640)
Logistic Regression Classifier	0.1	122 (111–132)	99.7 (99.6–99.9)	2.46 (2.23–2.69)	58.1 (54.4–61.8)	86.0 (79.3–92.6)	0.720 (0.693–0.747)
	0.2	89.3 (82.4–96.2)	99.7 (99.6–99.9)	3.20 (2.85–3.54)	69.6 (67.2–72.0)	82.9 (75.1–90.6)	0.762 (0.728–0.797)
	0.3	71.8 (66.4–77.2)	99.7 (99.6–99.8)	3.84 (3.39–4.29)	75.7 (73.9–77.6)	80.2 (72.1–88.3)	0.780 (0.742–0.817)
	0.4	59.1 (54.6–63.6)	99.7 (99.5–99.8)	4.43 (3.88–4.99)	80.1 (78.6–81.6)	76.9 (68.0–85.9)	0.785 (0.743–0.827)

	0.5	48.8 (45.0–52.6)	99.6 (99.5–99.7)	4.96 (4.30–5.61)	83.7 (82.4–85.0)	70.7 (61.8–79.6)	0.772 (0.729–0.815)
	0.6	40.5 (37.1–44.0)	99.4 (99.3–99.6)	4.94 (4.03–5.86)	86.4 (85.3–87.6)	57.4 (47.9–66.8)	0.719 (0.673–0.765)
Random Forest Classifier	0.1	111 (107–114)	100 (99.9–100)	3.07 (2.89–3.26)	62.2 (60.9–63.6)	99.3 (97.8–101)	0.808 (0.798–0.818)
	0.2	62.3 (59.6–65.1)	99.9 (99.8–100)	5.08 (4.72–5.44)	79.2 (78.2–80.1)	92.9 (87.7–98.1)	0.860 (0.836–0.885)
	0.3	32.4 (29.7–35.1)	99.6 (99.5–99.7)	7.48 (6.62–8.34)	89.4 (88.5–90.3)	69.3 (61.9–76.6)	0.794 (0.758–0.830)
	0.4	16.9 (14.3–19.5)	99.2 (99.1–99.3)	7.96 (5.96–9.96)	94.5 (93.6–95.4)	36.0 (28.3–43.6)	0.652 (0.615–0.689)
Gradient Boosting Classifier	0.1	62.1 (54.8–69.4)	99.8 (99.7–99.9)	5.21 (4.62–5.79)	79.2 (76.7–81.8)	87.9 (82.1–93.6)	0.835 (0.807–0.864)
	0.2	38.2 (34.5–41.9)	99.6 (99.5–99.8)	6.81 (5.98–7.63)	87.4 (86.2–88.7)	73.6 (65.3–81.8)	0.805 (0.765–0.845)
	0.3	26.7 (24.1–29.3)	99.5 (99.4–99.6)	8.29 (6.83–9.75)	91.3 (90.4–92.2)	61.9 (52.7–71.1)	0.766 (0.721–0.812)
	0.4	18.8 (16.4–21.3)	99.4 (99.3–99.5)	10.5 (7.53–13.5)	94.0 (93.1–94.8)	49.3 (40.6–57.9)	0.716 (0.674–0.759)
Voting Classifier	0.1	108 (102–114)	100 (99.9–100)	3.18 (2.96–3.41)	63.1 (61.0–65.2)	99.3 (97.8–100)	0.812 (0.799–0.824)
	0.2	68.7 (65.2–72.2)	99.9 (99.8–100)	4.57 (4.21–4.93)	76.9 (75.7–78.1)	91.7 (86.2–97.2)	0.843 (0.817–0.869)
	0.3	46.5 (43.4–49.5)	99.6 (99.5–99.7)	5.42 (4.76–6.08)	84.5 (83.5–85.6)	73.1 (64.4–81.7)	0.788 (0.746–0.830)
	0.4	31.6 (28.6–34.7)	99.5 (99.4–99.6)	7.29 (6.20–8.38)	89.6 (88.6–90.7)	64.0 (56.4–71.7)	0.768 (0.731–0.806)

**Classifiers**	**Cut-off threshold**	**Number of outcomes**	**Hypotension (N = 287)**				
			**Sensitivity**	**Specificity**	**PPV**	**NPV**	**AUC**
Pre–endoscop	1	228 (226–231)	92.9 (92.0–93.8)	10.6 (10.4–10.8)	21.3 (20.5–22.1)	85.2 (83.3–87.1)	0.584 (0.579–0.588)

y Rockall score	2	130 (127–132)	90.8 (90.2–91.4)	10.6 (9.80–11.4)	55.4 (54.5–56.2)	48.6 (44.7–52.5)	0.579 (0.574–0.583)
	3	84.4 (82.0–87.8)	90.5 (90.1–90.8)	10.7 (9.77–11.6)	70.9 (70.0–71.9)	31.7 (28.9–34.4)	0.574 (0.569–0.579)
	4	48.2 (46.4–50.0)	90.1 (89.9–90.4)	10.1 (8.94–11.2)	83.3 (82.6–84.0)	16.9 (15.0–18.7)	0.569 (0.564–0.574)
Glasgow–Blatchford Bleeding Score	10	158 (156–161)	94.5 (94.1–94.9)	13.4 (13.1–13.7)	47.1 (46.3–47.9)	74.7 (72.3–77.0)	0.588 (0.583–0.594)
	11	129 (127–132)	94.1 (93.7–94.5)	14.7 (14.3–15.1)	57.5 (56.7–58.3)	66.9 (64.4–69.4)	0.593 (0.588–0.599)
	12	101 (98.1–103)	92.5 (92.2–92.9)	14.3 (13.6–15.0)	66.8 (65.9–67.6)	50.7 (47.8–53.6)	0.592 (0.588–0.597)
	13	72.6 (70.5–74.7)	92.0 (91.7–92.4)	15.4 (14.4–16.3)	76.3 (75.7–77.0)	39.4 (36.5–42.3)	0.591 (0.586–0.596)
	14	40.2 (38.4–42.0)	92.2 (91.9–92.5)	22.3 (20.8–23.8)	88.0 (87.4–88.5)	31.7 (28.9–34.4)	0.592 (0.587–0.596)
Logistic Regression Classifier	0.1	249 (247–251)	98.8 (98.2–99.3)	11.2 (11.1–11.3)	14.8 (14.0–15.6)	98.1 (97.2–99.0)	0.564 (0.561–0.568)
	0.2	204 (200–207)	98.0 (97.5–98.5)	13.1 (12.9–13.3)	31.8 (30.6–32.9)	93.7 (92.0–95.3)	0.627 (0.621–0.634)
	0.3	166 (163–169)	97.2 (96.8–97.7)	15.0 (14.8–15.3)	45.6 (44.6–46.6)	87.8 (85.7–90.0)	0.667 (0.659–0.676)
	0.4	131 (128–134)	96.2 (95.9–96.6)	17.2 (16.7–17.6)	58.2 (57.1–59.2)	79.1 (76.8–81.4)	0.686 (0.675–0.697)
	0.5	98.2 (95.2–101)	95.1 (94.7–95.4)	19.4 (18.7–20.1)	69.5 (68.5–70.5)	66.9 (64.4–69.4)	0.682 (0.67–0.694)
	0.6	66.2 (63.5–69.0)	93.5 (93.1–93.8)	21.0 (20.0–22.1)	79.8 (79.0–80.7)	49.0 (46.0–51.9)	0.644 (0.63–0.658)
Random Forest Classifier	0.1	113 (110–117)	95.6 (95.2–96.1)	18.4 (17.6–19.1)	64.3 (63.0–65.7)	72.9 (69.9–75.9)	0.686 (0.671–0.701)

	0.2	39.6 (37.1–42.0)	92.5 (92.3–92.8)	25.0 (23.4–26.5)	88.5 (87.7–89.3)	34.5 (31.9–37.1)	0.615 (0.603–0.627)
	0.3	9.17 (8.03–10.3)	90.7 (90.6–90.9)	29.6 (24.3–34.8)	97.4 (97.0–97.9)	8.94 (7.26–10.6)	0.532 (0.523–0.541)
	0.4	1.60 (1.10–2.10)	90.2 (90.1–90.3)	46.7 (37.5–55.8)	99.5 (99.4–99.7)	1.41 (0.62–2.20)	0.505 (0.501–0.509)
Gradient Boosting Classifier	0.1	90.0 (86.0–93.9)	95.2 (94.8–95.5)	20.9 (20.1–21.8)	72.5 (71.2–73.9)	66.0 (62.8–69.1)	0.693 (0.679–0.706)
	0.2	33.5 (30.4–36.6)	92.8 (92.4–93.1)	30.1 (27.9–32.4)	90.9 (90.0–91.9)	35.1 (31.4–38.8)	0.630 (0.614–0.646)
	0.3	12.4 (10.9–13.9)	91.1 (90.9–91.3)	32.0 (28.8–35.1)	96.8 (96.3–97.2)	13.9 (11.9–15.9)	0.553 (0.544–0.562)
	0.4	3.17 (2.35–3.99)	90.3 (90.2–90.5)	38.3 (31.6–45.0)	99.1 (98.9–99.4)	3.23 (2.12–4.34)	0.512 (0.506–0.517)
Voting Classifier	0.1	200 (196–204)	97.6 (97.1–98.1)	13.1 (12.9–13.3)	32.9 (31.4–34.3)	92.4 (90.6–94.1)	0.626 (0.62–0.633)
	0.2	120 (117–124)	96.3 (95.9–96.7)	18.4 (17.8–19.0)	62.1 (61.0–63.2)	77.9 (75.3–80.5)	0.700 (0.687–0.713)
	0.3	61.0 (58.2–63.8)	94.2 (93.9–94.4)	25.1 (23.9–26.3)	82.3 (81.4–83.3)	53.4 (51.1–55.6)	0.679 (0.668–0.689)
	0.4	23.1 (21.3–24.9)	92.0 (91.8–92.2)	31.6 (29.1–34.1)	93.8 (93.3–94.4)	25.2 (22.9–27.4)	0.595 (0.584–0.606)

Classifiers	Cut-off threshold	Number of outcomes	Rebleeding within 7 days (N = 287)				
			**Sensitivity**	**Specificity**	**PPV**	**NPV**	**AUC**
Pre–endoscopy Rockall score	1	228 (227–230)	94.3 (93.4–95.3)	10.2 (10.0–10.5)	21.5 (20.8–22.1)	87.3 (85.2–89.5)	0.575 (0.570–0.580)
	2	130.0 (127.0–132.0)	91.7 (91.2–92.1)	10.5 (9.96–11.1)	55.6 (54.7–56.4)	50.7 (48.2–53.3)	0.572 (0.568–0.577)
	3	84.4 (82.3–86.5)	91.1 (90.9–91.4)	10.5 (9.75–11.2)	71.0 (70.2–71.9)	32.8 (30.6–35.0)	0.570 (0.565–0.574)

Glasgow–Blatchford Bleeding Score	4	48.2 (46.1–50.3)	91.1 (90.8–91.3)	11.3 (9.89–12.7)	83.6 (82.8–84.4)	20.1 (17.8–22.5)	0.567 (0.563–0.572)
	10	158 (155–161)	95.5 (95.0–96.0)	13.3 (12.8–13.7)	47.4 (46.3–48.4)	78.4 (75.8–80.9)	0.567 (0.563–0.571)
	11	129 (126–132)	94.5 (94.0–94.9)	14.0 (13.4–14.6)	57.4 (56.2–58.6)	67.2 (64.4–70.0)	0.570 (0.565–0.574)
	12	101 (97.4–104)	94.1 (93.8–94.5)	15.8 (15.0–16.5)	67.5 (66.4–68.7)	59.0 (56.2–61.7)	0.572 (0.568–0.576)
	13	72.6 (69.8–75.4)	93.4 (93.1–93.7)	17.4 (16.4–18.4)	77.0 (76.0–78.0)	47.0 (44.2–49.9)	0.574 (0.570–0.578)
	14	40.2 (38.6–41.8)	92.5 (92.2–92.8)	20.5 (18.8–22.3)	87.7 (87.1–88.4)	30.6 (28.0–33.2)	0.575 (0.571–0.579)
Logistic Regression Classifier	0.1	266 (263–269)	99.4 (98.9–99.9)	10.0 (9.93–10.1)	8.3 (7.27–9.33)	99.5 (99.0–99.9)	0.539 (0.534–0.544)
	0.2	219 (215–223)	97.7 (97.1–98.3)	11.5 (11.3–11.7)	25.7 (24.1–27.3)	93.8 (92.3–95.4)	0.598 (0.589–0.606)
	0.3	177 (173–181)	96.7 (96.1–97.3)	13.0 (12.7–13.4)	41.0 (39.6–42.5)	86.0 (83.4–88.7)	0.635 (0.623–0.648)
	0.4	134 (131–138)	95.6 (95.2–96.1)	15.0 (14.4–15.5)	56.2 (54.9–57.5)	74.8 (71.9–77.7)	0.655 (0.641–0.670)
	0.5	94.6 (91.4–97.8)	94.4 (94.0–94.8)	17.0 (16.2–17.8)	69.9 (68.7–71.0)	59.7 (56.8–62.5)	0.648 (0.633–0.663)
	0.6	60.3 (57.7–63.0)	93.2 (92.9–93.5)	18.8 (17.8–19.9)	81.2 (80.4–82.1)	42.3 (39.4–45.2)	0.618 (0.604–0.632)
Random Forest Classifier	0.1	111 (107–115)	95.0 (94.5–95.5)	16.1 (15.4–16.7)	64.3 (63.1–65.5)	66.7 (63.0–70.3)	0.655 (0.639–0.671)
	0.2	29.5 (27.1–32.0)	92.0 (91.7–92.4)	20.9 (18.5–23.3)	91.1 (90.3–91.8)	23.2 (19.9–26.6)	0.571 (0.556–0.587)
	0.3	4.71 (4.03–5.39)	91.2 (91.0–91.3)	41.2 (35.6–46.8)	98.9 (98.7–99.0)	6.49 (4.99–7.99)	0.527 (0.519–0.534)

	0.4	0.743 (0.502–0.984)	90.8 (90.7–90.9)	41.7 (–64.2–148)	99.9 (99.8–99.9)	1.38 (0.682–2.08)	0.506 (0.503–0.510)
Gradient Boosting Classifier	0.1	92.8 (86.7–98.9)	94.7 (94.2–95.3)	17.8 (16.7–18.8)	70.7 (68.7–72.7)	61.2 (56.4–66.0)	0.660 (0.640–0.679)
	0.2	29.9 (27.0–32.9)	92.2 (91.9–92.4)	22.7 (20.4–25.0)	91.1 (90.1–92.1)	24.7 (21.8–27.6)	0.579 (0.565–0.592)
	0.3	7.37 (6.2–8.54)	91.1 (90.9–91.3)	30.4 (24.4–36.3)	97.9 (97.5–98.3)	6.91 (5.11–8.72)	0.524 (0.515–0.533)
	0.4	1.63 (1.13–2.13)	90.8 (90.8–90.9)	48.0 (39.1–56.9)	99.6 (99.5–99.7)	2.12 (1.08–3.15)	0.509 (0.503–0.514)
Voting Classifier	0.1	212 (208–216)	97.5 (96.9–98.1)	11.7 (11.5–11.9)	28.3 (26.8–29.8)	92.6 (90.7–94.6)	0.605 (0.596–0.614)
	0.2	124 (120–127)	95.9 (95.4–96.4)	16.2 (15.6–16.9)	60.3 (59.2–61.5)	74.9 (71.6–78.1)	0.676 (0.660–0.692)
	0.3	55.9 (53.0–58.9)	93.2 (92.9–93.5)	19.8 (18.5–21.1)	82.8 (81.8–83.8)	41.1 (38.0–44.2)	0.619 (0.604–0.634)
	0.4	17.3 (15.8–18.8)	91.8 (91.6–92.0)	27.5 (24.1–31.0)	95.1 (94.6–95.7)	17.1 (14.9–19.2)	0.561 (0.550–0.572)

The means and 95% confidence intervals of scores are presented. The numbers in parentheses are confidence intervals. Total number of a test-set data is shown in parentheses after the table header, and the positive number of outcomes is presented in parentheses after the column name.

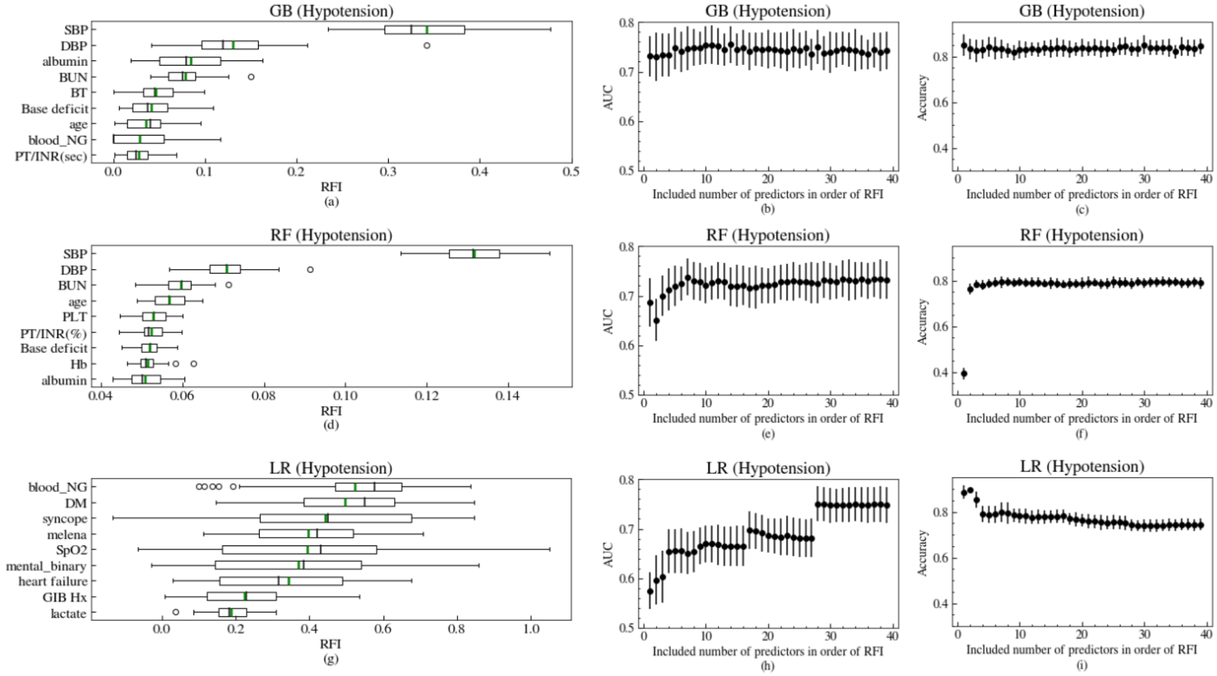

Figure S1. (**a**), (**d**), and (**g**) are the relative feature importance (RFI) of the top 9 features predicting hypotension by the three machine learning algorithms: LR (logistic regression with regularization), RF (random forest classifier), and GB (gradient boosting classifier), respectively. The black lines in boxes show the median of RFI and green lines show the mean of RFI. The AUC of the three models, with the included number of predictors in the descending order of RFI to predict hypotension, are plotted in (**b**), (**e**), and (**h**). The accuracies of the three models, according to the included number of predictors in the order of RFI to predict hypotension, are also plotted in (**c**), (**f**), and (**i**). The means of the AUC and accuracy are shown as black dots and the 1 standard deviation ranges from them are shown as black error-bars. See Table A1 for variable names.

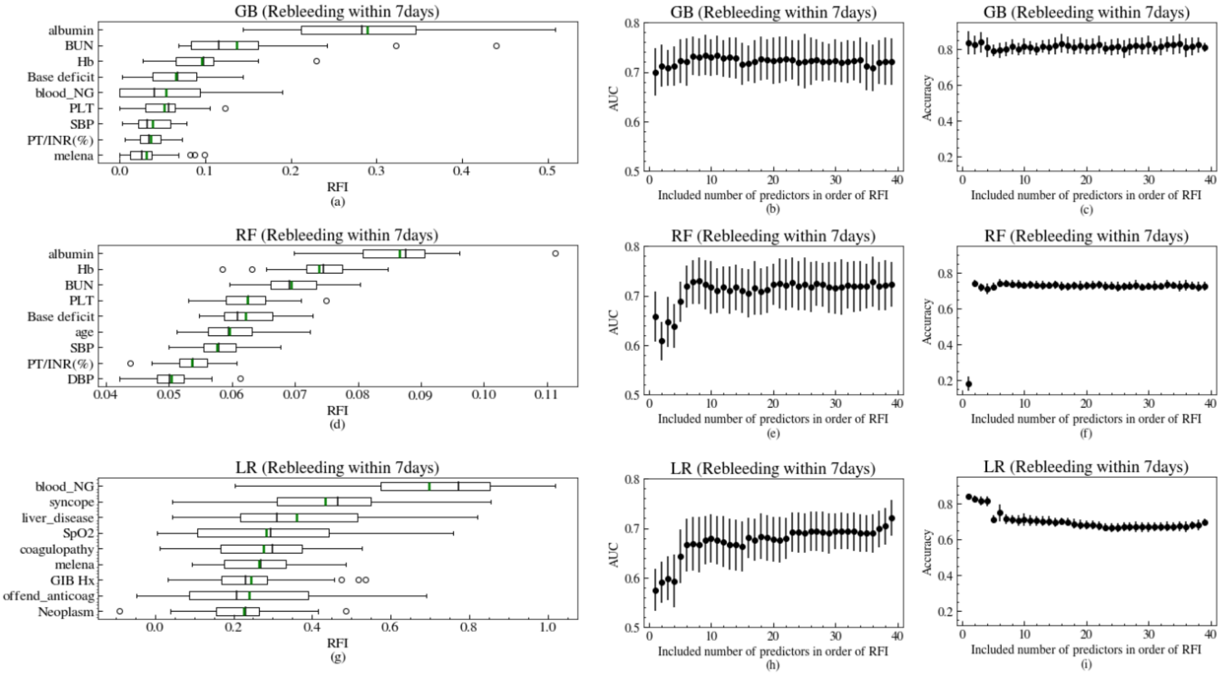

Figure S2. (**a**), (**d**), and (**g**) are the relative feature importance (RFI) of the top 9 features predicting rebleeding within 7 days by the three machine learning algorithms: LR (logistic regression with regularization), RF (random forest classifier), and GB (gradient boosting classifier), respectively. The black lines in boxes show the median of RFI and green lines show the mean of RFI. The AUC of the three models, with the included number of predictors in the order of RFI to predict rebleeding within 7 days, are plotted in (**b**), (**e**), and (**h**). The accuracies of the three models, according to the included number of predictors in the order of RFI to predict rebleeding within 7 days, are also plotted in (**c**), (**f**), and (**i**). The means of the AUC and accuracy are shown as black dots and the 1 standard deviation ranges from them are shown as black error-bars. See Table A1 for variable names.

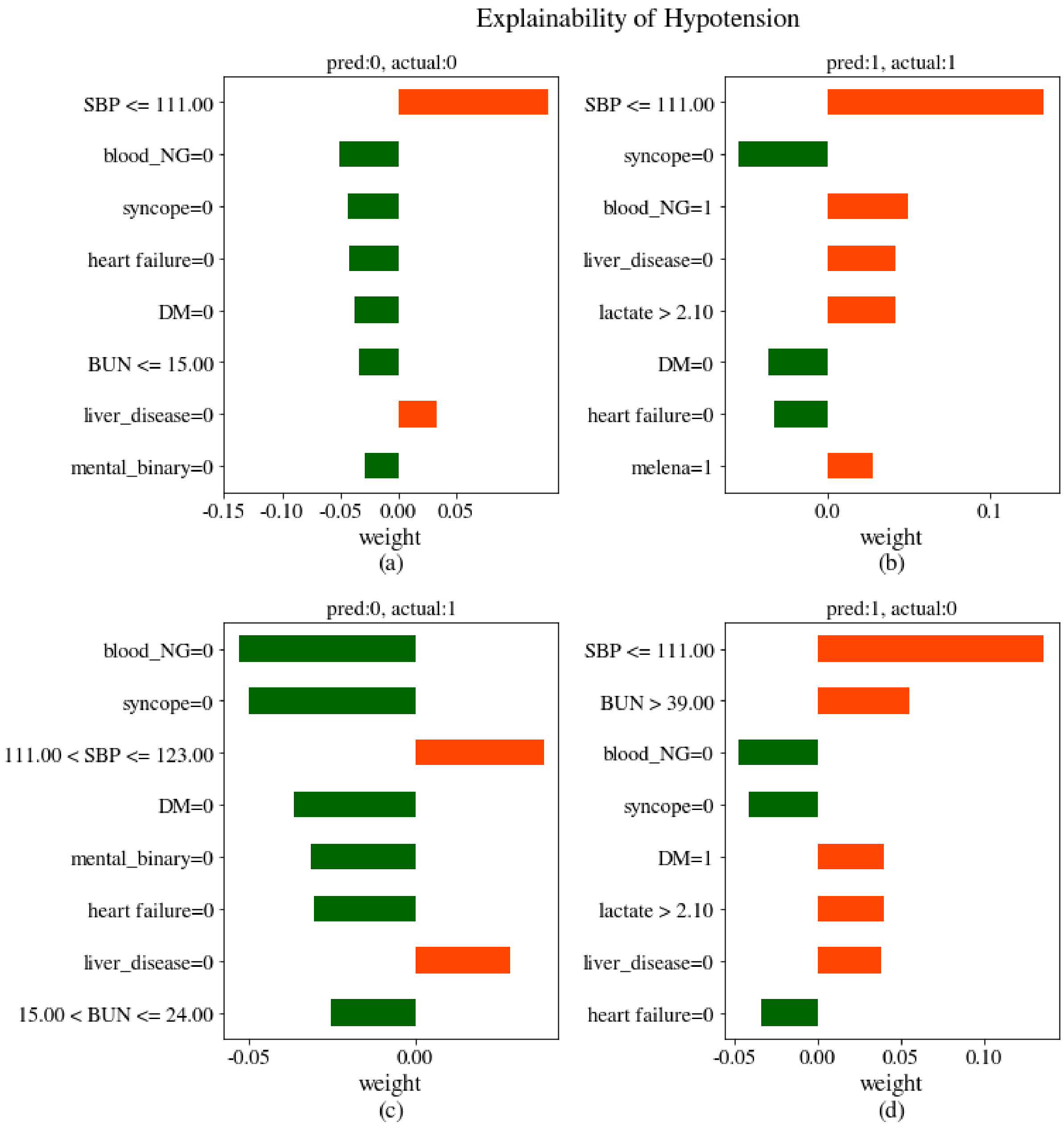

Figure S3. LIME (local interpretable model-agnostic explanation) of true-negative (**a**), true-positive (**b**), false-negative (**c**), and false-positive (**d**) cases for the VC when the outcome is hypotension. The orange bars are the variables that support the positive, and the green bars are the variables supporting the negative. The larger the absolute value of weight, the greater the effect of predicting outcome, hypotension. See Table A1 for variable names.

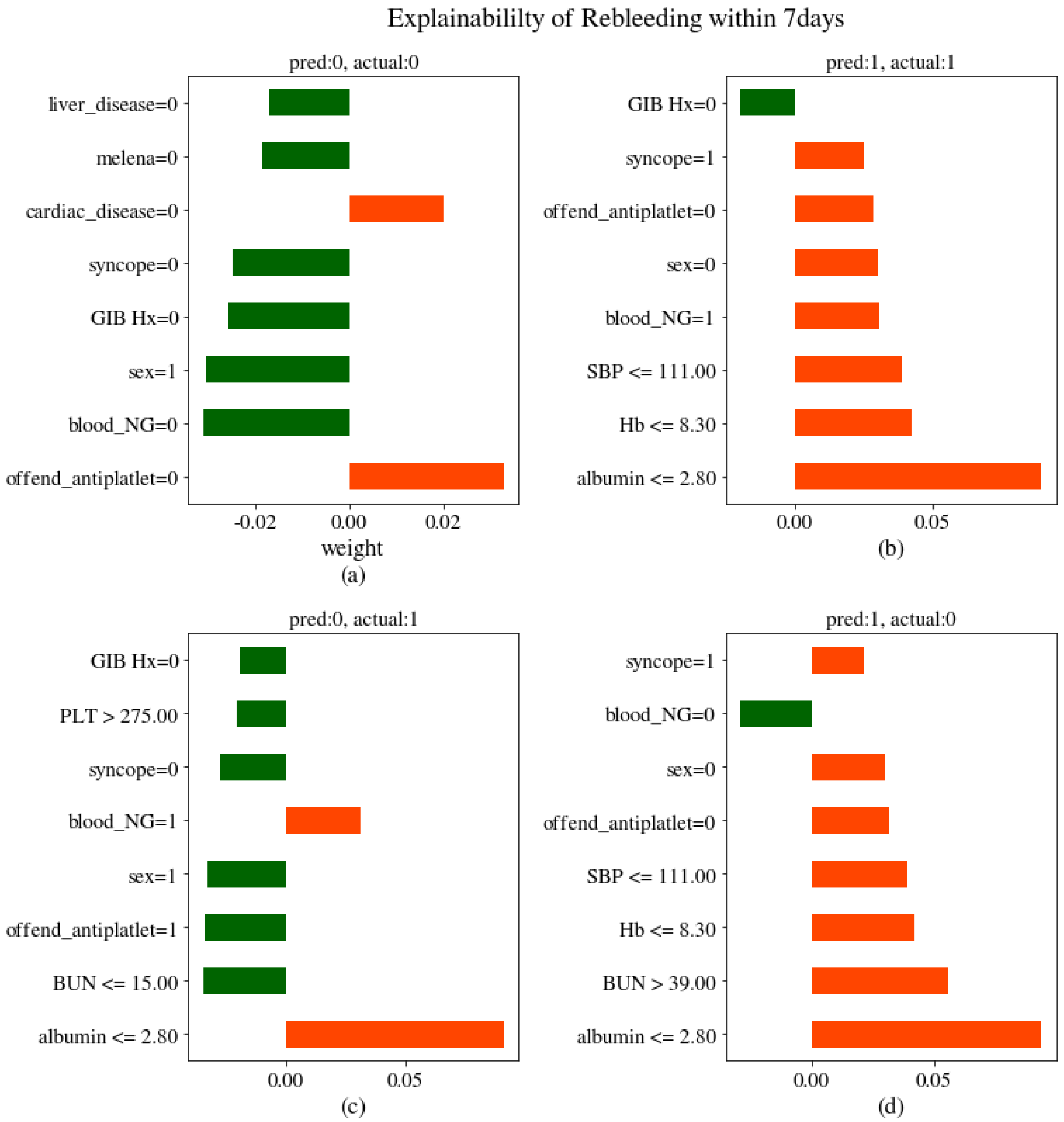

Figure S4. LIME (local interpretable model-agnostic explanation) of true-negative (**a**), true-positive (**b**), false-negative (**c**), and false-positive (**d**) cases for the VC when the outcome is rebleeding within 7 days. The orange bars are the variables that support the positive, and the green bars are the variables supporting the negative. The larger the absolute value of weight, the greater the effect of predicting outcome, rebleeding within 7 days. See Table A1 for variable names.
